# Phenylpyrroles: 30 Years, Two Molecules and (Nearly) No Resistance

**DOI:** 10.3389/fmicb.2016.02014

**Published:** 2016-12-16

**Authors:** Jaafar Kilani, Sabine Fillinger

**Affiliations:** ^1^UMR BIOGER, Institut National de la Recherche Agronomique, AgroParisTech, Université Paris SaclayThiverval-Grignon, France; ^2^Université Paris-Sud, Université Paris-SaclayOrsay, France

**Keywords:** fungicide, signal transduction, histidine kinase, resistance, fitness

## Abstract

Phenylpyrroles are chemical analogs of the natural antifungal compound pyrrolnitrin. Fenpiclonil, but mainly fludioxonil are registered against multiple fungal crop diseases since over 25 years for seed or foliar treatment. They have severe physiological impacts on the pathogen, including membrane hyperpolarization, changes in carbon metabolism and the accumulation of metabolites leading to hyphal swelling and burst. The selection and characterization of mutants resistant to phenylpyrroles have revealed that these fungicides activate the fungal osmotic signal transduction pathway through their perception by a typical fungal hybrid histidine kinase (HHK). The HHK is prone to point mutations that confer fungicide resistance and affect its sensor domain, composed of tandem repeats of HAMP motifs. Fludioxonil resistant mutants have been selected in many fungal species under laboratory conditions. Generally they present severe impacts on fitness parameters. Since only few cases of field resistance specific to phenylpyrroles have been reported one may suspect that the fitness penalty of phenylpyrrole resistance is the reason for the lack of field resistance.

## The Origin of Phenylpyrroles

Phenylpyrroles are chemical derivatives of pyrrolnitrin, a secondary metabolite produced by some bacteria from tryptophan (Floss et al., 1971). It was isolated for the first time from *Pseudomonas pyrrocinia* in the 1960s (Arima et al., 1965) and showed strong antifungal activity against various animal and plant pathogenic fungi even under greenhouse conditions. Pyrrolnitrin or pyrrolnitrin producing *Pseudomonas* (*e.g., P. fluorescens*) proved phytoprotecting efficiency against *Rhizoctonia solani, Alternaria* sp., *Fusarium* sp., *Verticillium dahliae*, and *Thielaviopsis basicola*. Its activity was found stable for 30 days in the soil (Howell and Stipanovic, 1979) but sensitive to light decomposition. Consequently, two synthetic analogs have been successfully developed by Ciba-Geigy AG (now Syngenta AG) in the 1980s and introduced in the market for seed treatment and foliar use (reviewed in Leadbitter et al., 1994).

**FIGURE 1:**
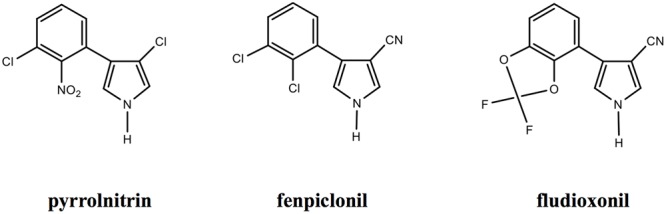
**Chemical structure of Pyrrolnitrin (Imanaka et al., 1965) and synthetic analogs (www.frac.org).** The chemical characteristics of phenylpyrroles are the phenyl ring with substitutions in positions 2 and/or 3 and the pyrrole ring with substitutions at position 3 (Corran et al., 2008).

Fenpiclonil and fludioxonil are 3-cyano-4-phenylpyrrol analogs of pyrrolnitrin with largely increased photo-stability and similar antifungal activity (reviewed in Corran et al., 2008). These compounds differ by the substitutions at positions 2 and 3 of the phenyl ring (**Figure 1**). Fenpiclonil (synthetized in 1984) was introduced in the market as seed-treatment in 1988 but rapidly superseded (1990) by the more stable and more active fludioxonil as foliar and seed-treatment (Leadbitter et al., 1994; Corran et al., 2008). To date, fludioxonil can be considered as major representative of the phenylpyrrole family of fungicides.

As non-systemic, surface fungicide, fludioxonil is registered for treatments at pre- and post-harvest stages on leaves, fruits and seeds. It has a principally prophylactic action against multiple fungal diseases provoked by ascomycetes or basidiomycetes. The list of crops registered for the use of fludioxonil and the associated pathogens (if known) is indicated in **Table 1**. Fludioxonil has no detectable activity on non-target organisms, such as baker’s yeast, men, plants, or animals (Gehmann et al., 1990).

**Table 1:** Crops and diseases registered for pre- or pos-tharvest treatment with phenylpyrroles.

Crop	Pathogens controlled	Reference
**Seed treatments and post-harvest uses**		
Almonds	*Coryneum beijerinckii, Monilinia spp.*	Gehmann et al., 1990
Avocado	*Dothiorella iberica*	Twizeyimana et al., 2013
	*Neofusicoccum australe*	
	*Neofusicoccum luteum*	
	*Neofusicoccum parvum*	
	*Phomopsis spp.*	
Barley	*Microdochium nivale*	Gehmann et al., 1990
	*Fusarium spp.*	
	*Ustilago hordei*	
	*Pyrenophora graminea*	
	*Cochliobolus sativus*	
Beans	*Rhizoctonia solani*	Olaya et al., 1994
	*Botrytis spp.*	Gehmann et al., 1990
Carrot	*n.i^∗^*	
Citrus fruit	*Penicillium digitatum*	Kanetis et al., 2007
Cotton	*Fusarium spp.*	Leroux et al., 1992;
	*Rhizoctonia solani*	Corran et al., 2008
	*Thielaviopsis basicola*	
Cucurbit vegetables	*n.i*	
Eggplant	*Botrytis spp.*	Corran et al., 2008
Flax seed	*n.i^∗^*	
Foliage of legume vegetables	*n.i^∗^*	
Ginseng	*n.i^∗^*	
Grapes	*Botrytis cinerea*	Gehmann et al., 1990
	*Glomerella cingulata*	
Grass (forage, fodder, hay)	*n.i^∗^*	
Jojoba	*n.i^∗^*	
Kiwifruit	*Botrytis cinerea*	Brigati et al., 2009
Lettuce	*Sclerotinia minor*	Gehmann et al., 1990
Maize	*Fusarium graminearum*	Gehmann et al., 1990
Tropical fruits	*n.i^∗^*	
Peanut	*Sclerotinia minor*	Gehmann et al., 1990;
	*Rhizoctonia solani*	Corran et al., 2008
Peas	*Ascochyta spp.*	Gehmann et al., 1990;
	*Fusarium spp.*	Corran et al., 2008
	*Peyronellaea pinodes*	
Pineapple	*n.i^∗^*	
Pistachio	*Alternaria spp.*	Ma et al., 2004
Pome fruit	*Penicillium spp., Botrytis cinerea*	Errampalli, 2004; Zhao et al., 2010
Pomegranate	*Botrytis cinerea*	Palou et al., 2007;
	*Alternaria spp.*	D’Aquino et al., 2010
	*Penicillium spp.*	
Potato	*Fusarium spp.*	Gehmann et al., 1990;
	*Helminthosporium solani*	Gachango et al., 2012
	*Boeremia exigua*	
	*Rhizoctonia solani*	
	*Alternaria solani*	
Rapeseed	*Leptosphaeria maculans*	Gehmann et al., 1990;
	*Alternaria brassicae*	Duan et al., 2013
	*Sclerotinia sclerotiorum*	
Rice	*Gibberella fujikuroi*	Gehmann et al., 1990
	*Rhizoctonia solani*	
	*Gaeumannomyces oryzinus*	
	*Cochliobolus miyabeanus*	
Rye	*Microdochium nivale*	Gehmann et al., 1990;
	*Urocystis occulta*	Corran et al., 2008
	*Monographella nivalis*	
Saﬄower	*n.i^∗^*	
Soybean	*Fusarium spp.*	Mueller et al., 1999;
	*Sclerotinia sclerotiorum*	Corran et al., 2008
	*Rhizoctonia solani*	
Stone fruits (apricots, peaches, nectarines, cherries, plums)	*Monilinia spp., Botrytis cinerea, Rhizopus spp.*	Gehmann et al., 1990; Förster et al., 2007
Strawberry	*Botrytis cinerea*	Gehmann et al., 1990;
	*Glomerella cingulata*	Taguchi et al., 2012
Sunflower	*n.i^∗^*	
Sweet potato	*Rhizopus stolonifer*	Edmunds and Holmes, 2009
Tomato	*Botrytis spp., Alternaria solani*	Gehmann et al., 1990
Tropical fruits	*n.i^∗^*	
Watercress	*n.i^∗^*	
Wheat	*Tilletia laevis*	Gehmann et al., 1990;
	*Microdochium nivale*	Corran et al., 2008
	*Fusarium spp.*	
	*Bipolaris sorokiniana*	
	*Phaeosphaeria nodorum*	
	*Monographella nivalis*	
**Terrestrial non-food uses**		
Turf	*Rhizoctonia solani*	Gehmann et al., 1990
	*Sclerotinia homeocarpa*	
	*Drechslera poae*	
	*Microdochium nivale*	
Ornamentals	*Rhizoctonia solani*	Gehmann et al., 1990

*^∗^n.i: not indicated (crops without associated pathogens were extracted from the registration review of fludioxonil).*

Phenylpyrroles inhibit all stages of fungal development, spore germination, germ-tube elongation, and mycelial growth (Leroux et al., 1992). The observed consequences are swollen hyphae with increased ramifications and apical lysis (Leroux, 1996) indicating that phenylpyrroles might act on the intra-hyphal turgor and cell wall biosynthesis (Lew, 2010).

## Effect of Phenylpyrroles on Target Fungi – Mode of Action

Jespers et al. (1994) observed extremely rapid intracellular accumulation of fenpiclonil in *Fusarium sulphureum* reaching its maximum in less than 1 min. Interestingly, the majority of the accumulated fenpiclonil can be washed off by water, suggesting that the phenylpyrrole penetrates the fungus through passive diffusion. The same study also showed that during the exposure to fenpiclonil the fungus accumulates the lipophilic cation tetraphenylphosphonium bromide (TPP^+^), independent of extracellular pH, indicating hyperpolarization of the plasma membrane and modification of the mitochondrial membrane potential (Jespers et al., 1994). Similar results have been observed with fludioxonil in *Neurospora crassa, i.e.*, the induction of hyperpolarization of the plasma membrane through eﬄux of H^+^ and influx of K^+^ leading to increased membrane potential (Lew, 2010).

Various authors have observed modifications in the intracellular accumulation of different metabolites (*e.g., F. sulphureum, N. crassa*). Exposure to high doses of fenpiclonil (over 10-fold EC_50_ concentrations) induces the accumulation of amino acids and monosaccharides (Jespers et al., 1993). Conversely the exposure to sub-lethal doses of phenylpyrroles seems to inhibit the incorporation of mono-saccharides into macromolecules (Jespers and De Waard, 1994) but also to stimulate biosynthesis and intracellular accumulation of glycerol and mannitol (Jespers and De Waard, 1995; Pillonel and Meyer, 1997). In order to precise the enzymatic step inhibited by phenylpyrroles, Jespers and De Waard (1995), studied the fate of radioactively labeled 2-deoxyglucose. 2-deoxyglucose can be phosphorylated as is glucose, but cannot be further metabolized. In the presence of fenpiclonil, [^14^C]-2-deoxyglucose accumulated intracellularly, while the intracellular concentration of [^14^C]-2-deoxyglucose-phosphate diminished indicating the inhibition of hexokinase activity during exposure of the mycelium to the phenylpyrrole. When the authors performed the same assay on crude mycelial extracts, they only observed a minor reduction of [^14^C]-2-deoxyglucose phosphorylation under high concentrations of fenpiclonil, withdrawing the cytoplasmic hexokinase as sole or direct target of fenpiclonil (Jespers and De Waard, 1995).

Pillonel and Meyer tested the inhibition of protein kinase activities in *N. crassa* by phenylpyrroles. They found that purified PK-III was inhibited by fenpiclonil and fludioxonil (Pillonel and Meyer, 1997). Although the concentration of phenylpyrroles required for PK-III inhibition was found similar to that of rat PKC-inhibition, *N. crassa* PK-III does not seem to be neither a Ca^2+^/calmodulin nor a cAMP regulated protein kinase (Judewicz et al., 1981; Ulloa et al., 1987). To some extend the inhibition of PK-III correlated with growth inhibition by fenpiclonil, but less by fludioxonil, raising the question if phenylpyrroles, especially fenpiclonil, directly inhibit PK-III activity. Given the data of Pillonel and Meyer, this hypothesis has never been retained nor validated, since the concentrations required to inhibit the purified enzyme (I_50_) were much higher (up to 100 times in the case of fludioxonil) than those needed to inhibit fungal growth (EC_50_). Either phenylpyrroles do not inhibit PK-III by itself, acting rather indirectly, or they may affect different cellular targets.

## From Phenylpyrroles to Osmotic Signal Transduction

Glycerol accumulation is a consequence specific to the exposure to phenylpyrrole, dicarboximide and aromatic hydrocarbon fungicides. The selection of laboratory generated mutants resistant to the three categories of fungicides in *B. cinerea* correlated with osmosensitivity (Leroux et al., 1992; Faretra and Pollastro, 1993). Also *N. crassa* osmosensitive mutants *os-1, os-2, os-4*, and *os-5* (Perkins et al., 1982) are resistant to dicarboximides, aromatic hydrocarbons, and phenylpyrroles (Fujimura et al., 2000; Zhang et al., 2002).

The corresponding genes and mutations have been cloned and identified in *N. crassa* and later in other fungi. The *os-1* gene encodes a class III HHK (Schumacher et al., 1997) whose mutations lead to fungicide resistance and osmosensitivity (Ochiai et al., 2001). The *os-2* gene on its turn encodes the osmosensing MAPK (Zhang et al., 2002), homologous to the MAPK of *Saccharomyces cerevisiae* involved in adaptation to high osmolarity named, high osmolarity glycerol, Hog1 (Hohmann, 2002). The fungicide resistant/osmosensitive phenotype of *os-2* mutants is due to non-sense mutations. Finally, *os-5* and *os-4* are the MAPKK and MAPKKK encoding genes, respectively (Fujimura et al., 2003). The Os-5, Os-4, and Os-2 elements are equivalent to the yeast osmotic ST cascade. Altogether these items suggest that the phenylpyrroles (and dicarboximides) target the osmotic ST cascade, in particular the class III HHK Os-1.

An additional argument for this hypothesis is the fact that the yeast *S. cerevisiae*, devoid of this class of HHK, is insensitive to phenylpyrroles and dicarboximides. The introduction of a class III HHK, orthologous to Os-1, leads to sensitivity to phenylpyrroles, dicarboximides and aromatic hydrocarbons in *S. cerevisiae* (Motoyama et al., 2005). These results are in favor of the class III HHK as direct target of phenylpyrroles (and dicarboximides).

**FIGURE 2:**
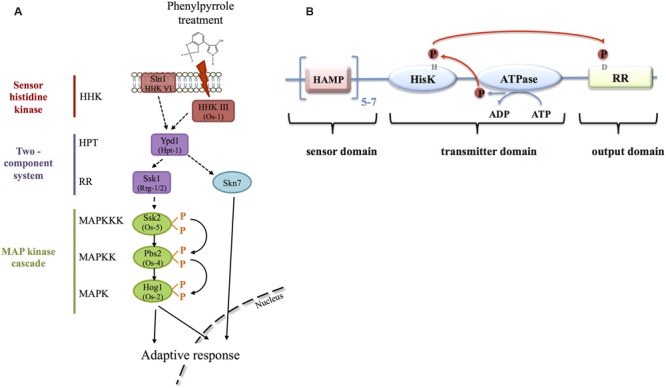
**Signal transduction of phenylpyrrole perception in ascomycetes. (A)** The fungicide signal is perceived by the HHK of class III, which transmits the signal to the osmotic MAPK cascade *via* the histidine-phosphate transfer protein (HPT) and the response regulator (RR). Phenylpyrrole treatment ultimately leads to MAPK-phosphorylation and activation of an adaptive response either through transcriptional activation in the nucleus or through the regulation of cytoplasmic proteins. The RR Skn7, under control of Ypd1, is also involved in the transcriptional regulation in response to phenylpyrrole treatment. The involvement of the HHK VI in the adaptation is less well documented. Protein names are those of *S. cerevisiae*, if different those of *N. crassa* are indicated in brackets. Full arrows indicate positive regulations, hashed arrows indicate either positive or negative regulations (different among fungal species), or direct interactions that remain to be demonstrated (reviewed in Bahn, 2008; Tanaka and Izumitsu, 2010; Jung et al., 2012; Herivaux et al., 2016). **(B)** Protein structure of class III fungal HHKs. The N-terminal domain, corresponding to the sensor domain is constituted of 5–7 tandem repeats of HAMP motifs. The C-terminal half is composed of the catalytic domains HK, ATPase and the RR. The conserved histidine residue in the HK domain is phosphorylated after hydrolysis of ATP by the ATPase. The phosphoryl group is then transferred to the conserved aspartate in the RR domain, which, ultimately, transfers the phosphoryl group to the HPT protein.

The possible mode of action is that fludioxonil, by binding to the class III HHK, mimics an osmotic stress through the activation of the Os-2/Hog1 MAPK (**Figure 2A**). This activation probably leads to multiple downstream reactions, such as activation of H+-ATPase, K+-influx and glycerol biosynthesis leading to increased intracellular turgor and membrane potential (Lew, 2010). Additional enzyme activities may be affected, *e.g*., hexokinase or sugar transporters (Jespers et al., 1994; Jespers and De Waard, 1995) that ultimately explain the phenotypes outlined above.

## Resistance to Phenylpyrroles

Until now only few cases of field resistance specific to fludioxonil have been reported; this despite the fact that for many fungal species (*N. crassa, B. cinerea, S. sclerotiorum, U. maydis, A. nidulans*,…) resistant strains could easily be obtained after mutagenesis and successive replication on fludioxonil supplemented medium (e.g., Avenot et al., 2005). These laboratory mutants display high resistance levels to phenylpyrroles, which is often associated with sensitivity to hyper-osmolarity and cross-resistance to dicarboximides and aromatic hydrocarbons (Ochiai et al., 2001; Leroux et al., 2002). In addition, most laboratory mutants, *e.g.*, in *B. cinerea* and *A. brassicicola*, display developmental defects and reduced pathogenicity (Avenot et al., 2005; Ajouz et al., 2010; Ren et al., 2016). Adversely, no fitness penalty was found associated with dicarboximide resistance (and phenylpyrrole sensitivity) in field strains (Oshima et al., 2002, 2006). Notably, in some fungal species, no developmental defect besides osmosensitivity was found associated with phenylpyrrole resistance (Motoyama et al., 2005; Luo et al., 2012).

Field strains cross-resistant to phenylpyrroles and dicarboximides have been isolated from *A. brassicicola, A. longipes*, and *A. alternata* populations (Dry et al., 2004; Iacomi-Vasilescu et al., 2004; Avenot et al., 2005; Luo et al., 2012; Avenot and Michailides, 2015; Malandrakis et al., 2015). No significant developmental defects could be detected in the *A. brassicicola* resistant mutants and only moderate osmosensitivity (Avenot et al., 2005; Iacomi-Vasilescu et al., 2008). However, phenylpyrrole resistance seems limited in *Alternaria* field populations (Avenot and Michailides, 2015; Malandrakis et al., 2015) indicating a potential fitness penalty not detected under controlled laboratory conditions.

Recently, fludioxonil resistant strains have been isolated from *B. cinerea* field populations in China, at low levels (<3%). They present the typical osmosensitivity and developmental defects of fludioxonil resistant laboratory mutants (Ren et al., 2016) raising the question of their capacity to compete with sensitive and fitter strains and the selective pressure of fungicide treatments on these particular populations. Globally, specific resistance to fludioxonil does not exist among gray mold populations maintaining the high efficiency of this fungicide (Walker et al., 2013; Fillinger and Walker, 2016). However, multi-drug resistant (MDR) phenotypes due to increased fungicide eﬄux affect sensitivity to fludioxonil (Kretschmer et al., 2009). Although MDR does not reach resistance levels sufficient to alter field efficacy of fungicides at their registered field rates, the MDR1h phenotype of *B. cinerea* group S strains leads to the highest resistance levels to fludioxonil reported for field isolates (Leroch et al., 2013) – besides the specific resistance reported from China (Ren et al., 2016) – and impacts fludioxonil efficacy at least in *in vitro* assays (Rupp et al., 2016).

## Fungal Histidine Kinases Linked to Phenylpyrrole Resistance

As mentioned above, mutations conferring resistance to phenylpyrroles and dicarboximides map to class III HHKs, although one cannot exclude the presence of mutations in other components of the osmotic ST cascades that have not been specifically searched for. HKs are ubiquitous, but typical fungal HHKs are absent from mammals and therefore constitute interesting targets for fungicide treatments. They are involved in cellular ST systems referred to as His-to-Asp phosphorelays. HHKs act as primary sensors for various environmental signals and initiate the adaptive response after autophosphorylation and subsequent phosphotransfer (reviewed by Bahn, 2008). Interestingly, the class III HHKs were shown to be cytoplasmic (Meena et al., 2010; Foureau et al., 2014), meaning that they sense fludioxonil intracellularly after its transmembrane diffusion.

Fungal HHKs are composed of the variable N-terminal sensor domain and the C-terminal domain, including the catalytic HK and ATPase domains that autophosphorylate the conserved histidine residue, in addition to the receiver domain with the cognate aspartate residue (reviewed in Jung et al., 2012; Herivaux et al., 2016) (**Figure 2**). A classification according to the structural components of the N-terminal domain and the peptide sequence around the conserved histidine residue attributed 16 classes of HHKs to fungi (Defosse et al., 2015). The number of HHK genes varies among species of the fungal kingdom from 1 to 21 HHKs (Catlett et al., 2003; Lavin et al., 2010; Defosse et al., 2015).

The HHKs involved in fludioxonil sensing are principally those belonging to class III (Ochiai et al., 2001; Avenot et al., 2005; Motoyama et al., 2005; Viaud et al., 2006; Dongo et al., 2009; Alberoni et al., 2010; Furukawa et al., 2012), but some data indicate a possible role in phenylpyrrole sensing of other HHKs. In *Candida lusitaniae* Chk1, the HHK of class VI, homologous to the osmosensing HHK Sln1 of *S. cerevisiae*, interferes with phenylpyrrole sensitivity (Chapeland-Leclerc et al., 2007). In the *Cryptococcus neoformans*, Tco2, a basidiomycete specific dual HK is also involved in fludioxonil sensitivity (Bahn et al., 2006). If the action of these HHKs is direct or indirect through the HOG pathway remains to be established.

The N-terminal domain of class III HHKs is characterized by 5–7 tandem repeats of an approximately 50-amino acid alpha-helical region, conserved among several signaling proteins and named HAMP domain (IPR003660). HAMPs have been extensively studied in bacterial sensor proteins where they play an active role in the intramolecular ST from the transmembrane sensor domain to the cytoplasmic kinase domain. It has been suggested that the HAMP domain regulates the phosphorylation of homodimeric sensor proteins by transmitting the conformational changes in the ligand-binding domains to the C-terminal signaling kinase domains (Aravind and Ponting, 1999; Klose et al., 2014; Schultz et al., 2015). This model is supported by genetic and biochemical studies (Zhou et al., 2009; Matamouros et al., 2015).

Histidine kinase, adenylate cyclase, methyl accepting proteins, phosphatases modules do not have strict sequence conservation, but a canonical coiled coil structure. HAMP subunits have two 16-residue amphiphilic helices (AS1, AS2) joined by a 14- to 15-residue connector segment. AS1 and AS2 have a seven-residue repeat pattern with hydropic residues at the first and forth position, respectively (Parkinson, 2010). Rotation after signal perception is proposed to constitute the basic mechanism of HAMP mediated transmembrane signaling in bacteria (Airola et al., 2010, 2013; Klose et al., 2014).

The 5–7 repeats of HAMP modules and the cytoplasmic localization do not allow a simple transposition of the bacterial structure-function model to explain the mechanism of ST in fungal HKs. The number of repeat units varies across fungal species. Using *S. cerevisiae* as heterologous host the role of HAMP domains in ST has been investigated. In the case of *Debaryomyces hansenii* class III HHK, HAMP deletion and yeast two hybrid studies led to the proposal of a functional model to explain the transduction of the hyperosmolarity or fludioxonil signal involving the five HAMP domains of the DhNik1 protein (Meena et al., 2010; Furukawa et al., 2012): The correct order of the HAMP domains is essential; HAMP1-3, 5 are essential for kinase activity, but HAMP4 is essential for the regulation of the HHK in response to a signal through its interaction with HAMP5. Using this approach, the authors showed that DhNik1 in the heterologous host *S. cerevisiae* has a functional kinase activity under standard conditions inhibiting the phosphorylation of the MAPK Hog1. Hyperosmolarity or fludioxonil inhibit DhNik1 activity leading to Hog1 activation. The interaction between two HAMP domains (HAMP4 and HAMP5) is essential for HHK inhibition. The authors also showed in the yeast model, that a constitutive active form of DhNIK1 confers resistance to fludioxonil. Among point mutations of *N. crassa* mutants displaying low resistance to fludioxonil (Ochiai et al., 2001), at least one of these mutations leads to a constitutive active form of the class III HK, conferring fludioxonil resistance to *S. cerevisiae* (Furukawa et al., 2012).

Mutations in fungal class III HHKs conferring resistance to phenylpyrroles and cross-resistance to dicarboximides generally induce phenotypes similar to deletion mutants (Viaud et al., 2006; Fillinger et al., 2012). They localize within or between the HAMP domains of the HHKs (Oshima et al., 2002; Alberoni et al., 2010; Fillinger et al., 2012; Firoz et al., 2015), while others are frameshift or non-sense mutations (Ochiai et al., 2001; Iacomi-Vasilescu et al., 2004; Duan et al., 2014; Ren et al., 2016). Altogether these results are in agreement with the hypothesis that in most cases loss-of-function mutations are responsible for fludioxonil resistance in plant pathogenic fungi (mainly laboratory mutants; reviewed in Defosse et al., 2015), but mutations leading to modified function or even constitutively active HHK may exist as well, probably at very low frequencies. Due to its essential role in many biological processes including pathogenicity (Viaud et al., 2006; Herivaux et al., 2016), loosing a class III HHK might explain the absence of fludioxonil field resistance in most plant pathogenic fungi.

## Conclusion

Thirty years after their introduction in the fungicide market, the large spectrum phenylpyrroles still hide some mysteries. Although all characterized resistance mutations have been mapped to class III HHK genes, the corresponding protein has never been demonstrated as phenylpyrrole target. It has been shown that fludioxonil, the nearly unique representative of this class of fungicides, activates the osmosensing MAPK in divers fungi (Kojima et al., 2004; Yoshimi et al., 2005; Bahn et al., 2006; Hagiwara et al., 2007; Segmuller et al., 2007) which may be its real mode of action. One may hypothesize that this permanent stimulation of the hyper-osmolarity response *via* MAPK activation induces the observed pleiotropic phenotypes and, consequently, fungal death.

Another mystery is the absence (or low abundance) of fludioxonil field resistance. To our knowledge field isolates displaying specific resistant to fludioxonil have been detected only in *Alternaria* sp. (Iacomi-Vasilescu et al., 2004) and, very recently in *B. cinerea* (Ren et al., 2016). In most cases fludioxonil resistance due to mutations in the HHK gene seems to induce a strong fitness penalty; *e.g*., extremely reduced sporulation, osmosensitivity, loss of pathogenicity, etc. (Ziogas et al., 2005; Viaud et al., 2006; Ajouz et al., 2011; Malandrakis et al., 2015), definitely counter-selecting fludioxonil resistance. In the case of *A. brassicicola*, the absence of evident developmental defects in some fludioxonil resistant field isolates (Iacomi-Vasilescu et al., 2004), might be due to compensatory mutations in a given genetic background. Nevertheless spreading of these strains might be limited under field conditions due to some yet undetected defect. Therefore it might be suspected that evolution of fludioxonil resistance in fungal populations is strongly limited, unless additional mutations compensating the fitness penalty may arise and be selected. After 30 years of phenylpyrroles the chances to select such multiple mutations seem limited; otherwise they would have already appeared.

Another question raised while writing this review is the absence of alternative structural analogs of pyrrolnitrin that could have been produced by the chemical companies; an astonishing fact since fenpiclonil and fludioxonil have a large spectrum of activity, high efficiency and are not really facing resistance problems. Were similar components synthesized, but did not show comparable efficiency or stability? Are there problems with other phenylpyrroles that fenpiclonil or fludioxonil do not face? Is synthesis too complicated or expensive? At Ciba Geigy, among the multiple analogs tested, fenpiclonil and fludioxonil were the only molecules with the required properties for efficient fungicides (Leadbitter et al., 1994; Pillonel and Meyer, 1997) and their registration, suggesting potential problems in synthesis, activity, stability, and/or toxicity issues of other analogs.

With increasing resistance problems against medical antifungal compounds, class III HHKs have been considered as potential drug targets also against human fungal pathogens (Bahn et al., 2005; Nemecek et al., 2006; Chapeland-Leclerc et al., 2007; Randhawa et al., 2016), especially since the target is specific of the pathogen. Phenylpyrroles could constitute the next generation of clinical antifungals, but for this sector, we are not aware of any compound in clinical testing, although pyrrolnitrin served as lead structure for pharmaceutical research (e.g., Umio et al., 1969). The absence of clearly characterized molecular interaction between phenylpyrroles and class III HHKs may explain the absence of clinical analogs of phenylpyrroles or other inhibitors of these proteins. Inhibition studies of known protein kinases may help the identification of new antifungal molecules [*e.g*., in the model fungus *N. crassa* (Pillonel, 2005), the plant pathogenic fungus *Ustilago maydis* (Tueckmantel et al., 2011; Grutter et al., 2012), the human pathogenic fungi *C. neoformans* and *C. albicans* (Tsuda et al., 2011; Lee et al., 2015)], but phenylpyrrole-analogs do not figure among the tested molecules.

From a fundamental point of view, the activation of the osmotic ST pathway by phenylpyrroles also raises questions. Do phenylpyrroles share the same ST elements as an hyperosmolarity treatment? If they bind to the class III HHK, what are the interacting domains? Do they differ from those recognizing hyperosmolarity (or dicarboximides)? Is resistance to fludioxonil conferred to by HHK loss-of-function mutations only, or are some of the mutations dominant active forms? These last questions require a thorough analysis of the ST processes after perception of phenylpyrroles, which may ultimately help understanding their mode of action.

## Author Contributions

JK and SF designed the plan of the manuscript. JK wrote sections 1–4, SF wrote sections 5 and 6 and completed the review.

## Conflict of Interest Statement

The authors declare that the research was conducted in the absence of any commercial or financial relationships that could be construed as a potential conflict of interest.
